# Chloroform in Parturition

**Published:** 1892-11

**Authors:** 


CHLOROFORM IN PARTURITION.—In the *Med. and Surg. Reporter*, Dr. L. Ridgeway Barber discusses ably the question of administering chloroform in labor to women who have organic heart disease. He concludes that chloroform by inhalation can and will, if properly administered, save the lives of parturient females, suffering from organic disease, when death seems imminent from overstimulation of its ganglia through reflex nervous action. Organic heart disease, then, does not preclude the use of chloroform in labor, but rather is a condition calling for its careful administration.

